# Correlation Between Plasma Proteomics and Adverse Outcomes Among Older Men With Chronic Coronary Syndrome

**DOI:** 10.3389/fcvm.2022.867646

**Published:** 2022-04-19

**Authors:** Yu-Lun Cai, Ben-Chuan Hao, Jian-Qiao Chen, Yue-Rui Li, Hong-Bin Liu

**Affiliations:** ^1^Department of Cardiology, The Second Medical Center and National Clinical Research Center for Geriatric Diseases, Chinese PLA General Hospital, Beijing, China; ^2^Medical School of Chinese PLA, Beijing, China; ^3^Beijing Key Laboratory of Chronic Heart Failure Precision Medicine, Beijing, China

**Keywords:** proteomics, chronic coronary syndrome (CCS), prognosis, aging, geriatrics

## Abstract

**Background:**

Chronic coronary syndrome (CCS) is a newly proposed concept and is hallmarked by more long-term major adverse cardiovascular events (MACEs), calling for accurate prognostic biomarkers for initial risk stratification.

**Methods:**

Data-independent acquisition liquid chromatography tandem mass spectrometry (DIA LC-MS/MS) quantitative proteomics was performed on 38 patients with CCS; 19 in the CCS events group and 19 in the non-events group as the controls. We also developed a machine-learning-based pipeline to identify proteins as potential biomarkers and validated the target proteins by enzyme-linked immunosorbent assay in an independent prospective cohort.

**Results:**

Fifty-seven differentially expressed proteins were identified by quantitative proteomics and three final biomarkers were preliminarily selected from the machine-learning-based pipeline. Further validation with the prospective cohort showed that endothelial protein C receptor (EPCR) and cholesteryl ester transfer protein (CETP) levels at admission were significantly higher in the CCS events group than they were in the non-events group, whereas the carboxypeptidase B2 (CPB2) level was similar in the two groups. In the Cox survival analysis, EPCR and CETP were independent risk factors for MACEs. We constructed a new prognostic model by combining the Framingham coronary heart disease (CHD) risk model with EPCR and CETP levels. This new model significantly improved the C-statistics for MACE prediction compared with that of the Framingham CHD risk model alone.

**Conclusion:**

Plasma proteomics was used to find biomarkers of predicting MACEs in patients with CCS. EPCR and CETP were identified as promising prognostic biomarkers for CCS.

## Introduction

The Guidelines for chronic coronary syndrome (CCS) were announced at the annual meeting of the European Society of Cardiology (ESC) in August 2019 (1). The ESC updated the “Guidelines for Treatment of Stable Coronary Artery Diseases (SCAD)” released in 2013 (2) and defined the concept of CCS. This changed the previous inherent concept of SCAD and reflected the current deeper understanding of the pathophysiological mechanism of coronary artery disease. Traditionally, the term SCAD was used to describe CCS which often shaped the disease as “stable.” Although CCS is often “relatively stable” compared with acute coronary artery disease, the underlying pathophysiological state can become “unstable” at any time, causing plaque rupture or erosion, leading to acute thrombosis. Therefore, the risk of future cardiovascular events in patients with CCS can be different; hence, predicting major adverse cardiovascular events (MACEs) will have great clinical significance for patients with CCS.

The purpose of genomics is to collectively characterize and quantify all genes, and transcriptomics refers to the study of gene transcription in cells and the regulation of transcription regulation at the overall level. However, in recent years, many researchers have found that the results for proteins are often not highly consistent with those for genomes or transcriptomes (3). This is partly because the products of transcription or translation are usually metabolized or modified, thereby changing the downstream protein abundance (4). Proteins are key regulators of many biological processes, and are directly related to the occurrence of many diseases and their clinical prognosis (5). Proteomics approaches have become increasingly mature, from the discovery of a single biomarker for early disease to the comprehensive characterization of protein abundance profiles of specific diseases. Indeed, some diseases are affected by more than one biological pathway. The advent of high-throughput proteomics has made research on such processes possible, and the study of differentially expressed proteins (DEPs) has provided insights into the molecular mechanisms of many human diseases (6–8). Currently, liquid chromatography tandem mass spectrometry (LC-MS/MS) is the main tool used to analyze whole proteomes, and it has been applied in studies of cardiovascular diseases, including the recently redefined CCS.

In this study, we performed a proteomics analysis to discover potential biomarkers of CCS, used machine learning methods to screen the identified biomarkers (9, 10), and validated the selected biomarkers in an independent prospective cohort. Two proteins were identified as new biomarkers for predicting the risk of adverse cardiovascular events in patients with CCS.

## Materials and Methods

**FIGURE 1:**
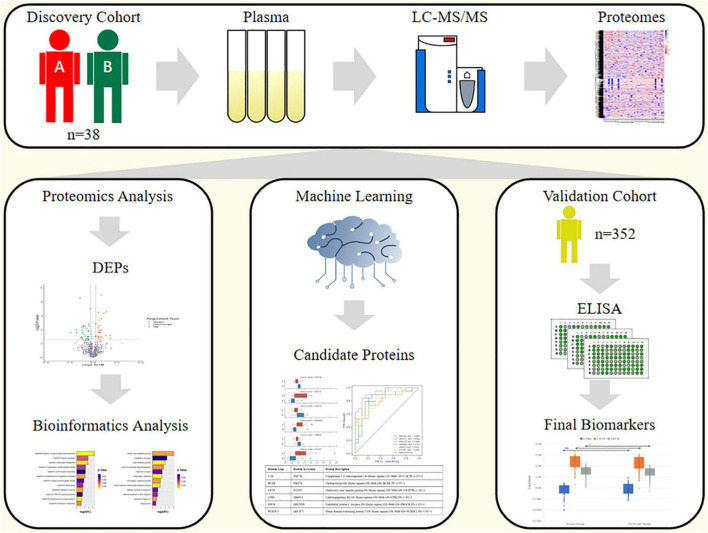
Study design.

The overall design of this study is shown in Figure 1. The discovery cohort was a retrospective cohort. We selected 38 patients who had undergone a physical examination conducted at the People’s Liberation Army (PLA) General Hospital from April to July 2015. The inclusion criteria were: (1) patients who were asymptomatic or had stable symptoms within 1 year after onset of acute coronary syndromes; (2) patients who were stable more than 1 year after initial diagnosis or revascularization regardless of symptoms; (3) patients with angina pectoris, suspected vasospasm, or microvascular disease; and (4) Males aged more than 65 years old. The exclusion criteria were: (1) patients with severe heart failure; (2) patients with CHD in the acute phase; and (3) patients with other diseases that made them unsuitable for this study. According to whether MACEs (including cardiovascular related death, non-fatal myocardial infarction, unstable angina, and heart failure) occurred until June 2019, the participants were divided into a CCS events group (Group A, *n* = 19) as the cases and a non-events group (Group B, *n* = 19) as the controls for the proteomics analysis. The validation cohort was an independent prospective cohort that included 352 patients who were recruited from those who had undergone a routine physical examination at the PLA General Hospital from April to July 2017. The inclusion and exclusion criteria were the same as those for the discovery cohort above. This cohort was followed up until April 2021. All methods were carried out in accordance with relevant guidelines and regulations. All experimental protocols were approved by Ethics Board of the Chinese PLA General Hospital. All informed consent was obtained from all subjects and/or their legal guardian(s).

### Blood Sampling

Plasma samples were collected after fasting 12 h. The samples were stored at −80°C with ethylenediaminetetraacetate until analysis.

### Proteomics Analysis

Quantitative proteomics analysis was performed using liquid chromatography tandem mass spectrometry (LC-MS/MS) to identify potential protein biomarker candidates among those proteins differing in abundance between event group and non-event group.

We used an integrated approach involving data-independent acquisition (DIA) strategy, HPLC fractionation to quantify the dynamic changes of the whole proteome.

#### Protein Extraction

Firstly, the cellular debris of plasma sample was removed by centrifugation at 12,000 *g* at 4°C for 10 min. Then, the supernatant was transferred to a new centrifuge tube. The top 12 high abundance proteins were removed by Pierce™ Top 12 Abundant Protein Depletion Spin Columns Kit (Thermo Fisher). Finally, the protein concentration was determined with BCA kit according to the manufacturer’s instructions.

#### Trypsin Digestion

For digestion, the protein solution was reduced with 5 mM dithiothreitol for 30 min at 56°C and alkylated with 11 mM iodoacetamide for 15 min at room temperature in darkness. The protein sample was then diluted by adding 100 mM TEAB to urea concentration less than 2 M. Finally, trypsin was added at 1:50 trypsin-to-protein mass ratio for the first digestion overnight and 1:100 trypsin-to-protein mass ratio for a second 4 h-digestion.

#### HPLC Fractionation

The tryptic peptides were fractionated into fractions by high pH reverse-phase HPLC using Agilent 300Extend C18 column (5 μm particles, 4.6 mm ID, 250 mm length). Briefly, peptides were first separated with a gradient of 8–32% acetonitrile (pH 9.0) over 60 min into 60 fractions. Then, the peptides were combined into 18 fractions and dried by vacuum centrifuging.

#### Data-Independent Acquisition—Liquid Chromatography Tandem Mass Spectrometry Analysis

The iRT kit was added to all the samples according to manufacturer’s instructions. The tryptic peptides were dissolved in solvent A (0.1% formic acid, 2% acetonitrile), directly loaded onto a home-made reversed-phase analytical column (25-cm length, 100 μm i.d.). Peptides were separated with a gradient from 4 to 32% solvent B (0.1% formic acid in 90% acetonitrile) over 114 min, and climbing to 80% in 3 min then holding at 80% for the last 3 min, all at a constant flowrate of 450 nL/min on an EASY-nLC 1200 UPLC system (Thermo Fisher Scientific). The separated peptides were analyzed in DDA mode by Q Exactiveis™ HF-X (Thermo Fisher Scientific) with a nano-electrospray ion source.

The separated peptides were analyzed in Q Exactive™ HF-X (Thermo Fisher Scientific) with a nano-electrospray ion source. The full MS scan resolution was set to 120,000 for a scan range of 385–1200 m/z. The data acquisition was performed in DIA mode. Each cycle contains one full scan followed by 70 DIA MS/MS scans with predefined precursor m/z range. The higher-energy collisional dissociation (HCD) fragmentation was performed at a normalized collision energy (NCE) of 27%. The fragments were detected in the Orbitrap at a resolution of 15,000. Fixed first mass was set as 200 m/z. Automatic gain control (AGC) target was set at 5E5.

#### Data Analysis

Spectral library generation: the resulting DDA data were processed using MaxQuant search engine (v.1.6.6.0). Tandem mass spectra were searched against the human SwissProt database (20,387 entries) concatenated with reverse decoy database. Trypsin/P was specified as cleavage enzyme allowing up to 2 missing cleavages. The mass tolerance for precursor ions was set as 20 ppm in First search and 4.5 ppm in Main search, and the mass tolerance for fragment ions was set as 0.02 Da. Carbamidomethyl on Cys was specified as fixed modification. Acetylation on protein N-terminal and oxidation on Met were specified as variable modifications. FDR was adjusted to <1%. The false discovery rates of the PSMs and proteins were set to less than 1%.

Data-independent acquisition data analysis: all DIA data were analyzed in Skyline (v 4.1.0). The DDA search results were imported to Skyline to generate the spectral library, and the retention times were aligned to iRT reference values. Transition settings: precursor charges were set as 2, 3, 4, 5, ion charges were set as 1, 2. The ion match tolerance was set as 0.02 Da. Six most intense fragment ions from the spectral library were selected for each precursor. Decoy generation was based on shuffled sequences, and the FDR was estimated with the mProphet approach and set to 1%. Relative quantification of proteins was performed using MSstats package.

### Bioinformatics Analysis

For the Gene Ontology (GO) and Kyoto Encyclopedia of Genes and Genomes (KEGG) analyses, we used the two-tailed Fisher’s exact test to determine the significance of the functional enrichment of the DEPs against all the identified proteins. A corrected *p*-value < 0.05 was considered significant.

### Machine Learning-Based Selection of Biomarkers

#### Construction of Voting Classifier

We used three machine learning classification algorithms, Logistic Regression, Support Vector Machine, and Random Forest, as the base classifiers (Supplementary Figure 1). On the basis of these classifiers, we built a voting classifier. When a new sample had to be assigned to a category, each base classifier was used to predict the probability that the new sample belonged to a particular category. The final classification result was determined by the weighted value of the predicted probability of each category by all three base classifiers. This is an integrated method that used the Voting Classifier model in Python.

#### Feature Ranking

Each sample was represented by a feature vector composed of numerous expression data. To quantify the ability of these expression features to distinguish different samples, we performed univariate feature analysis using a variance test to calculate the correlation between each feature and the sample category one by one. In this way, the ability of each feature to distinguish the sample category was obtained and the score and the corresponding *p*-value was calculated. The expression features were sorted according to the calculated *p*-value, and used in the subsequent analysis.

#### Accuracy Evaluation Index

To compare the difference between the category predicted by the model and the actual sample category, we calculated an accuracy index using the Matthews coefficient value as an indicator of the accuracy of the predictive power of the model as


(1) Matthews coefficient=(TP×TN-FP×FN)/√⁢[(T⁢P+F⁢P)⁢(T⁢P+F⁢N)⁢(T⁢N+F⁢N)⁢(T⁢N+F⁢P)]

where, TP, TN, FP, and FN are true positive, true negative, false positive, and false negative, respectively.

#### Feature Selection

We plotted the calculated accuracy index results against the number of features in the expression feature subset as the incremental feature selection curve. When the Matthews coefficient reached the maximum value, we considered the expression feature subset corresponding to the model as the optimal expression feature subset.

#### Cross-Validation

The expression feature subset and sample category were used as input of the Voting Classifier model, and 10-fold cross-validation was used to calculate the prediction accuracy of the local optimal expression feature subset for a sample. The results were expressed by the receiver operating characteristic curve (ROC). This is a dynamic validation that reduced the impact of data partitioning.

### Statistics Analysis

Data are presented as numbers and frequencies for categorical variables and as means ± SD for continuous variables. Baseline characteristics were compared using the Chi-square test for categorical variables and analysis of variance test for continuous variables. The effect of the candidate biomarkers was evaluated using a Cox proportional hazards model, and *p*-values were calculated using the log-rank test. The ROC was compared using a *z*-test (DeLong’s method) between the classic Framingham CHD risk model alone and the Framingham CHD model combined with the candidate biomarkers EPCR and CETP. The Framingham CHD model included age, sex, total cholesterol, high-density lipoprotein cholesterol (HDL-C), systolic blood pressure, current smoking, and diabetes status as confounding factors. *p*-Value of <0.05 was considered to indicate statistically significant difference for all the analyses. The statistical analyses were performed using SPSS (version 23.0), STATA (version 12.0), and MedCalc Software.

### Data and Code Availability

The datasets generated and/or analyzed during the current study are available in the PRIDE repository.^1^ Project ID: PXD029473.

## Results

### Baseline Characteristics

The baseline clinical data of the discovery and validation cohorts are shown in Table 1. The discovery cohort comprised 19 cases (including 2 cardiovascular death, 4 non-fatal myocardial infarction, 12 unstable angina, and 1 heart failure) and 19 controls, with an average follow-up time of 47.6 months. In the validation cohort, after an average of 41.6 months of follow-up, 86 patients (24.3%) had MACEs (including 9 cardiovascular death, 12 non-fatal myocardial infarction, 57 unstable angina, and 8 heart failure). Age, fibrinogen, D-dimer, glycosylated hemoglobin, and NT-proBNP (N-terminal-pro-brain natriuretic peptide) were significantly higher in the events group than they were in the non-events group. Current smoking, diastolic blood pressure, and HDL-C were significantly higher in the non-events group than they were in the events group.

**TABLE 1:** Baseline clinical and laboratory characteristics of the study patients.

	Discovery cohort		Validation cohort	
		
	Event (*n* = 19)	No event (*n* = 19)	Event (*n* = 86)	No event (*n* = 266)
Age, years	79.13 ± 12.12	78.39 ± 6.55	83.78 ± 9.66	80.41 ± 9.15*
Waistline (cm)	91.00 ± 10.18	90.20 ± 7.45	91.75 ± 12.20	92.85 ± 11.64
BMI (kg/m^2^)	23.89 ± 1.94	23.70 ± 1.66	24.26 ± 2.90	24.72 ± 3.41
Current smokers, *n* (%)	1 (5.3)	1 (5.3)	5 (5.8)	38 (14.3)*
Hypertension, *n* (%)	14 (73.7)	16 (84.2)	86 (75.6)	194 (72.9)
Diabetes mellitus, *n* (%)	12 (63.2)	9 (47.4)	35 (40.7)	97 (36.5)
Stroke, *n* (%)	3 (15.8)	3 (15.8)	10 (11.6)	28 (10.5)
Systolic pressure (mmHg)	129.84 ± 17.98	137.73 ± 15.31	134.30 ± 16.50	132.92 ± 17.54
Diastolic pressure (mmHg)	64.42 ± 11.49	67.15 ± 11.85	65.72 ± 9.82	68.43 ± 9.59*
Fibrinogen (g/L)	3.46 ± 0.66	3.46 ± 0.52	3.52 ± 0.63	3.35 ± 0.61*
D-dimer (mmol/L)	0.66 ± 0.62	0.26 ± 0.23	0.96 ± 1.79	0.64 ± 0.65*
Total cholesterol (mmol/L)	3.97 ± 0.76	3.78 ± 0.67	3.97 ± 0.77	4.03 ± 0.79
Triglyceride (mmol/L)	1.44 ± 0.79	1.19 ± 0.35	1.42 ± 0.75	1.26 ± 0.70
HDL-C (mmol/L)	1.24 ± 0.28	1.28 ± 0.38	1.25 ± 0.40	1.41 ± 0.50*
LDL-C (mmol/L)	2.55 ± 0.67	2.25 ± 0.55	2.49 ± 0.67	2.44 ± 0.70
HBA1c (%)	6.27 ± 1.14	6.24 ± 0.86	6.36 ± 1.17	6.10 ± 0.79*
NT-proBNP (pg/ml)	225.04 ± 230.82	201.21 ± 181.24	344.21 ± 499.52	213.06 ± 400.00*

BMI, body mass index; HDL-C, high-density lipoprotein cholesterol; LDL-C, low-density lipoprotein cholesterol; HBA1c, hemoglobin A1c; NT-proBNP: N-terminal-pro-brain natriuretic peptide. *p-value < 0.05.

### Proteomics Analysis

Quality control results for the proteomics data are shown in Supplementary Figure 2. The length distribution showed that 70% of the identified peptides were 7–20 amino acids long, which is consistent with the general rules of trypsin enzymatic hydrolysis and HCD. Peptides shorter than 5 amino acids cannot be effectively identified, and peptides longer than 20 amino acids are not suitable for HCD because of their high mass and charge. The distribution of peptides per protein showed that there were more than two peptides for most proteins. In general, proteins that have multiple corresponding specific peptides increase the precision and accuracy of the quantification results. The mass distribution of the proteins showed that the mass was relatively well-distributed, indicating that there was no significant molecular weight bias for proteins during sample preparation. These results confirm that the overall proteomics data meet the quality control requirements.

A total of 5,480 peptides were identified in the mass spectrum. By comparing the peptides back to proteins, we identified a total of 1,120 proteins, of which 783 were quantified.

We used the proteomic data to identify signatures of MACEs by analyzing the plasma proteins that underwent significant fold changes (FCs) between Group A and Group B (FC > 1.2 or FC < 0.8; unpaired two-sided Welch’s *t*-test; *p* < 0.05). As shown in the volcano map (Supplementary Figure 3), a total of 57 DEPs were identified under this condition.

### Functional Analysis of the Differentially Expressed Proteins

**FIGURE 2:**
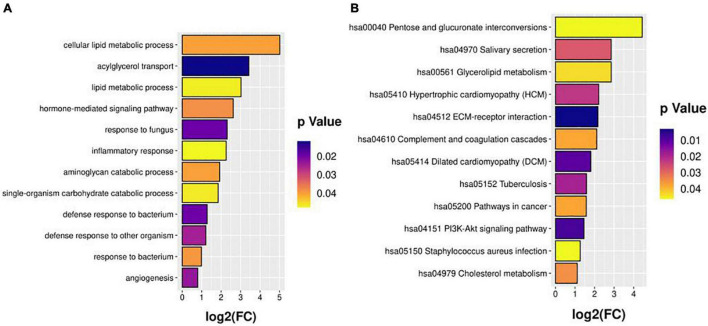
GO **(A)** and KEGG **(B)** pathway enrichment analyses.

The DEPs were annotated with GO terms and assigned KEGG pathways for the functional enrichment analyses (Figure 2). The highly enriched processes included cellular lipid metabolic process, acylglycerol transport, inflammatory response, response to bacterium, glycerolipid metabolism, hypertrophic cardiomyopathy, complement and coagulation cascades, and PI3K-Akt signaling pathway. These analyses pinpointed specific pathways (lipid metabolic, coagulation, and inflammation) that may operate in the events group compared with the non-events group, and highlighted CCS-related pathways for further functional investigation.

### Machine Learning-Based Selection of Biomarkers for Prognosis of Chronic Coronary Syndrome

We used the plasma proteomic data of the discovery cohort and developed a series of algorithms to identify potential biomarker combinations to classify CCS cases. The feature analysis was used to rank the expression features based on scores and *p*-values, as shown in Supplementary Figure 4. To visualize the ranking results of the protein expression characteristics, we plotted a ranking histogram of the top 30 features with the highest scores (Supplementary Figure 5).

**FIGURE 3:**
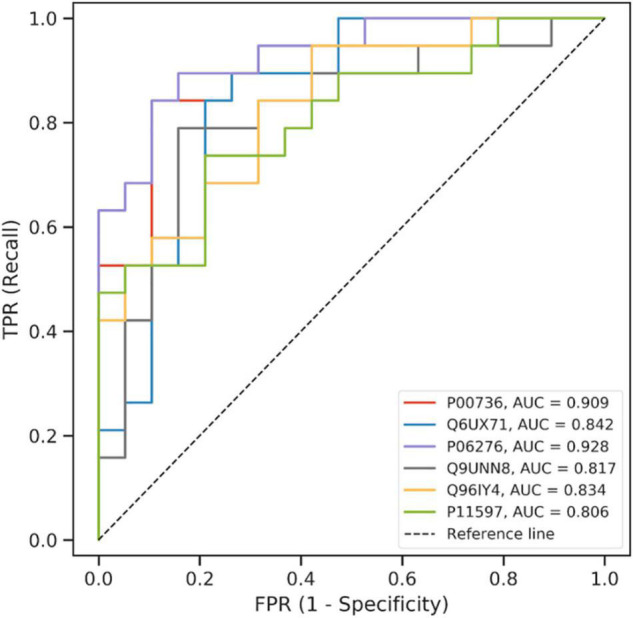
ROC curve of the model using the candidate proteins in machine learning.

The incremental feature selection curve (Supplementary Figure 6) shows that when the top eight ranked features were selected, the Matthews coefficient value of the model reached the maximum for the first time. These eight features were analyzed further. Next, we selected the protein combinations with the highest area under the curve (AUC) value from the 10-fold cross-validation. The ROC curve (Figure 3) showed that the AUC of the first six proteins in the final trained model were >0.8. These six proteins were identified as candidate proteins and the detailed information is shown in Supplementary Table 1. The distribution of these six proteins between the events and non-events groups was significantly different as shown in the box plots in Supplementary Figure 7.

### Validation of the Biomarkers of Different Chronic Coronary Syndrome Outcomes

**FIGURE 4:**
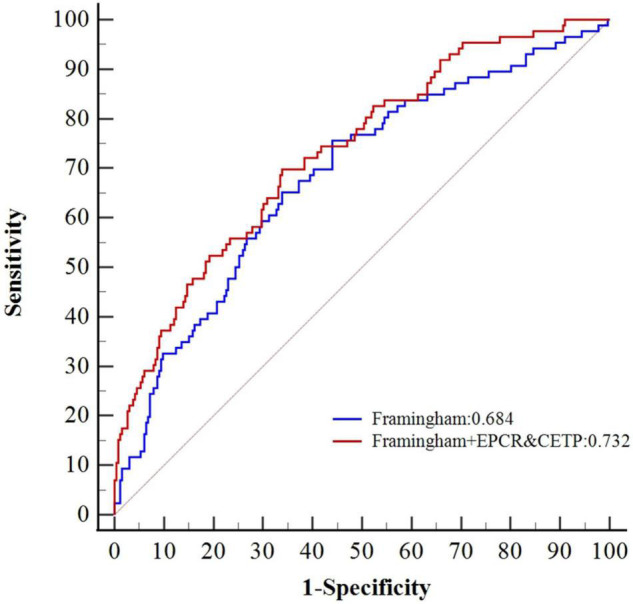
Comparative ROC curves of Framingham CHD risk model combined with CETP and EPCR.

By combining the results of the DEP selection, GO and KEGG pathway analyses, machine learning, previous knowledge and clinical relevance, we selected three proteins EPCR (Q9UNN8), CPB2 (Q96IY4), and CETP (P11597) as the target proteins for validation. The expression levels of the three proteins are shown in Supplementary Figure 8. EPCR and CETP levels at admission were significantly higher in the CCS events group than in the control non-events group, whereas the CPB2 levels were similar. The Cox survival analysis showed that EPCR and CETP were risk factors for MACEs (Table 2). After correcting for the confounding factors in the Framingham CHD risk model, EPCR and CETP were still found to be independent risk factors for MACEs. Additionally, a new prognostic model was constructed by combining the Framingham CHD risk model with the candidate biomarkers EPCR and CETP. This new model significantly improved the C-statistics for MACE prediction compared with that of the Framingham CHD risk model alone (AUC 0.732 vs. 0.684, *p* < 0.05) (Figure 4). These results showed that EPCR and CETP were independent risk factors for MACEs in patients with CCS, and combined with the classic Framingham model, EPCR and CETP provided better prediction metrics than the Framingham model alone.

**TABLE 2:** Relation of target proteins and MACEs in univariate and multivariate survival analysis.

	Univariate models		Multivariate models	
		
	HR (95% CI)	*p*-Value	HR (95% CI)	*p*-Value
CPB2	0.843 (0.690–1.030)	0.096	0.824 (0.676–1.004)	0.055
CETP	1.063 (1.005–1.125)	0.034	1.058 (1.000–1.120)	0.048
EPCR	1.086 (1.021–1.155)	0.008	1.075 (1.013–1.142)	0.017

*CPB2, carboxypeptidase B2; CETP, cholesteryl ester transfer protein; EPCR, endothelial protein C receptor. Multivariate model included age, sex, total cholesterol, high-density lipoprotein cholesterol, systolic blood pressure, current smoking, and diabetes status. CETP is calculated as per 100 mmol/L and EPCR is calculated as per 10 mmol/L.*

## Discussion

We performed a series of studies on the plasma proteins of patients with CCS, followed by global proteomic mass spectrometry identification, machine learning-based selection of biomarkers, and enzyme-linked immunosorbent assay (ELISA) for prospective validation of expanded samples. First, we used discovery mass spectrometry to quantify thousands of different proteins without the need for previous knowledge, and thus identify proteins not previously associated with CCS. After protein relative quantification between the CCS events and non-events groups, the classification power was evaluated by machine learning-based selection. On the basis of the results and clinical relevance, we identified three proteins, endothelial protein C receptor (EPCR), carboxypeptidase B2 (CPB2), and cholesteryl ester transfer protein (CETP) as candidate biomarkers. Finally, in the independent validation cohort, we found that EPCR and CETP were better than CPB2 for predicting MACEs in patients with CCS by ELISA and, when combined with the Framingham coronary heart disease (CHD) risk model, they improved the risk prediction beyond the Framingham CHD risk model alone.

Cardiovascular disease is one of the leading causes of death worldwide (11). One of the main risks of developing cardiovascular disease is vascular endothelial dysfunction (12). In a healthy state, endothelial cells maintain a balanced hemostatic state by producing procoagulants and anticoagulants, as well as proinflammatory and anti-inflammatory cytokines. In the disease state, endothelial cells are activated and exert procoagulant and proinflammatory effects. Endothelial cell dysfunction leads to thrombosis and coagulation imbalance (13). The new international guidelines consider that CHD is a dynamic process of atherosclerotic plaque accumulation and changes in coronary circulatory function. Plaques can show the following trends: gradually increased instability or even rupture, stability maintained for a long time, and gradual shrinking. The composition of plaques also continues to change. However, about one-third of patients with a cardiovascular disease have angina pectoris, but have no obstructive coronary artery disease (14). Therefore, patients with CCS can have a relatively stable period, but the relatively stable vascular environment and circulatory function may become unstable because of inflammatory reactions, vulnerable plaques, and abnormal lipid metabolism (15). Therefore, “stability” is only temporary and relative, not absolute. The clinical evaluation and management of such seemingly “stable” patients with CCS is of great significance to improve the prognosis. In this study, we found EPCR and CETP were closely related to vascular endothelial homeostasis.

There are two variants of EPCR: mEPCR (membrane EPCR), which is present on endothelial cell membranes, and soluble EPCR (soluble EPCR), which circulates in the blood (16). Protein C is a vitamin K-dependent serine protease that is synthesized mainly in the liver and circulates in the plasma. Protein C binds to mEPCR with high affinity, and is converted to activated protein C (APC) by the thrombin–thrombomodulin complex on the surface of endothelial cells through a limited proteolytic process (17). The mEPCR variant can bind to APC and plays important roles in anticoagulation, anti-inflammatory, cell protection (anti-apoptosis), protecting endothelial barrier function, and promoting neovascularization (18–22). When activated by the thrombin–thrombomodulin complex, APC dissociates from the membrane-bound receptor mEPCR, and functions as an anticoagulant by inactivating coagulation factors Va and VIIIa (23). When APC is combined with mEPCR, it shows strong anti-inflammatory and cytoprotective activities. The cytoprotective signal activity of APC is mediated by protease activated receptor 1 (PAR1) on endothelial cells bound by mEPCR (24). The APC–mEPCR complex relies on the anti-inflammatory activity of PAR1 to mediate the inhibition of inflammatory gene expression, including *c-Fos* and *FosB*, which belong to the activator protein 1 (AP-1) family. Protective signals can also inhibit the release of inflammatory cytokines (such as IL-1β, IL-6, and tumor necrosis factor-α) and the nuclear translocation of NF-κB, and downregulate the expression of genes that encode endothelial cell adhesion proteins (such as ICAM1, VCAM1, and E-selectin), thereby restricting the penetration of white blood cells through the vascular system, protecting the endothelial barrier function, and inhibiting inflammation (25). Conversely, the sEPCR variant detaches from the cell membrane surface through shedding and enters the circulation. Possible reasons for shedding include a systemic inflammatory response and vascular endothelial damage (26). The sEPCR plays a negative competitive role in blood circulation. It binds protein C and APC with similar affinity and inhibits protein C activation on the endothelium. It also inhibits the anticoagulant activity of APC by blocking the binding of APC to phospholipids. It is the sEPCR variant in blood circulation that was tested in this study. Elevated levels of sEPCR can disturb vascular homeostasis, promote coagulation, aggravate inflammation, and accelerate endothelial cell apoptosis, which is associated with increased risk of thrombosis (27).

Cholesteryl ester transfer protein is a plasma protein secreted by the liver. It is one of the most effective endogenous regulators of plasma HDL-C, which protects the cardiovascular system in many ways (28). It can promote the transfer of cholesterol ester from HDL-C to apolipoprotein B (ApoB). In addition to removing excess cholesterol from the arterial wall, HDL-C can also inhibit lipid oxidation, restore endothelial function, and exert anti-inflammatory and anti-apoptotic effects (29, 30). CETP leads to a net reduction of HDL-C in plasma, which increases the risk of atherosclerosis development. Some large randomized controlled trials have studied the effects of CETP inhibitors on lipid metabolism and MACEs, but the results are inconsistent (31–33). Possible reasons include incomplete function of the CETP inhibitors and side effects of drugs. A study explored whether CETP was related to atherosclerosis through its role in HDL-C and low-density lipoprotein metabolism. In a case–control study of 50 patients with coronary atherosclerosis and 50 controls, no significant difference was detected in the lipid profiles between the two groups, even though the serum CETP level of the case group was significantly higher than that of control group (34). This finding indicated that CETP may have atherogenic effects. However, no further studies have reported the long-term adverse prognosis risk of CHD. In our study, we included both case–control and prospective cohorts, which more fully illustrated the relationship between CETP and the poor prognosis of patients with CCS.

On the other hand, several studies are designed to provide the genomic or metabolomic background of CHD (35–37). All these studies, once combined together, might provide essential evidence for the efficient risk-stratification of patients with CHD.

To our knowledge, this is the first study to evaluate the relationship between plasma proteomics and the prognosis of patients with CCS. This study was layered involving three different methods, and the results are accurate and reliable. In addition, we analyzed more cases with longer follow-up times than most of the other studies. Our study has some limitations. First, the discovery and validation cohorts were from a single center and had relatively fewer older adult female patients. Therefore, to reduce bias, only male patients were included in this study. Second, we identified 57 DEPs that could predict the occurrence of MACEs, which may provide a rich biomarker pool for CCS, but further refinement of the diagnostic biomarkers is needed. Third, whether EPCR and CETP will act as prognostic predictors of CCS caused by other etiologies needs further study.

## Conclusion

We performed plasma proteomics analysis to find biomarkers for predicting MACEs in patients with CCS. We identified EPCR and CETP as independent risk factors for MACEs. The Framingham CHD risk model combined with EPCR and CETP was found to be a high-performance prognostic model for CCS. EPCR and CETP may be associated with vascular homeostasis, involving lipid metabolism, and inflammatory, coagulation, and cell protection processes. Further investigations are needed to understand the specific mechanisms involved.

## Data Availability Statement

The datasets presented in this study can be found in online repositories. The names of the repository/repositories and accession number(s) can be found in the article/Supplementary Material.

## Ethics Statement

The studies involving human participants were reviewed and approved by the Ethics Board of the Chinese PLA General Hospital. The patients/participants provided their written informed consent to participate in this study.

## Author Contributions

Y-LC and H-BL designed the protocol. Y-LC drafted the manuscript. H-BL supervised the patient recruitment and study procedures. Y-LC, B-CH, J-QC, and Y-RL conducted the study procedures. All authors carried out study procedures, critically revised the manuscript for important intellectual content, and approved the final manuscript.

## Conflict of Interest

The authors declare that the research was conducted in the absence of any commercial or financial relationships that could be construed as a potential conflict of interest.

## Publisher’s Note

All claims expressed in this article are solely those of the authors and do not necessarily represent those of their affiliated organizations, or those of the publisher, the editors and the reviewers. Any product that may be evaluated in this article, or claim that may be made by its manufacturer, is not guaranteed or endorsed by the publisher.

## Footnotes

1
http://proteomecentral.proteomexchange.org
