# Supplementary material for: Correlation Between Plasma Proteomics and Adverse Outcomes Among Older Men With Chronic Coronary Syndrome
Source: Front Cardiovasc Med. 2022 Apr 19;9:867646. doi: 10.3389/fcvm.2022.867646 (PMC9062975; doi:10.3389/fcvm.2022.867646)
Supplement: Supplementary file 1 [file Data_Sheet_1.DOCX]

Supplementary figure S1. Flow chart of Machine Learning


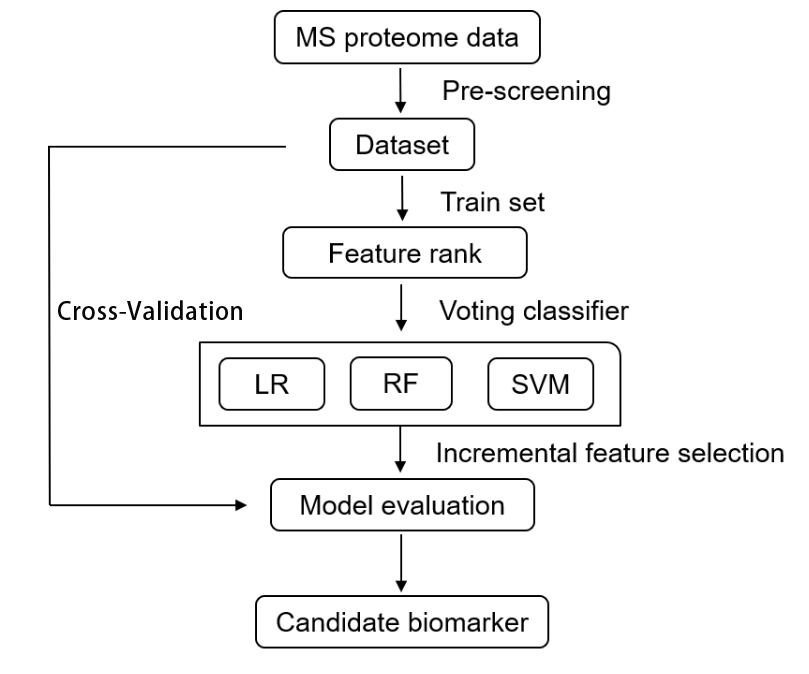


Supplementary figure S2. Quality control of the proteomics

1. Length distribution of all identified peptides


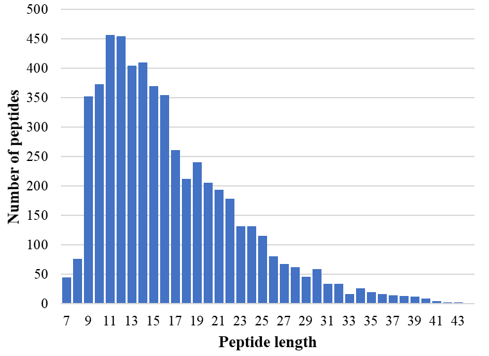


1. Distribution diagram of the number of peptides


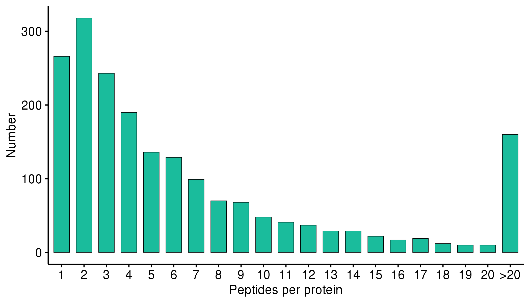


1. Distribution diagram of protein mass


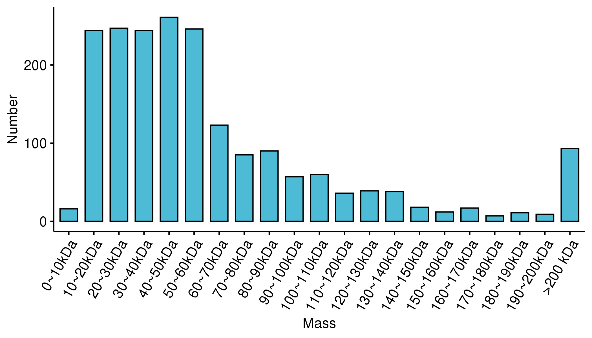


Supplementary figure S3.Volcano plot of differentially expressed proteins


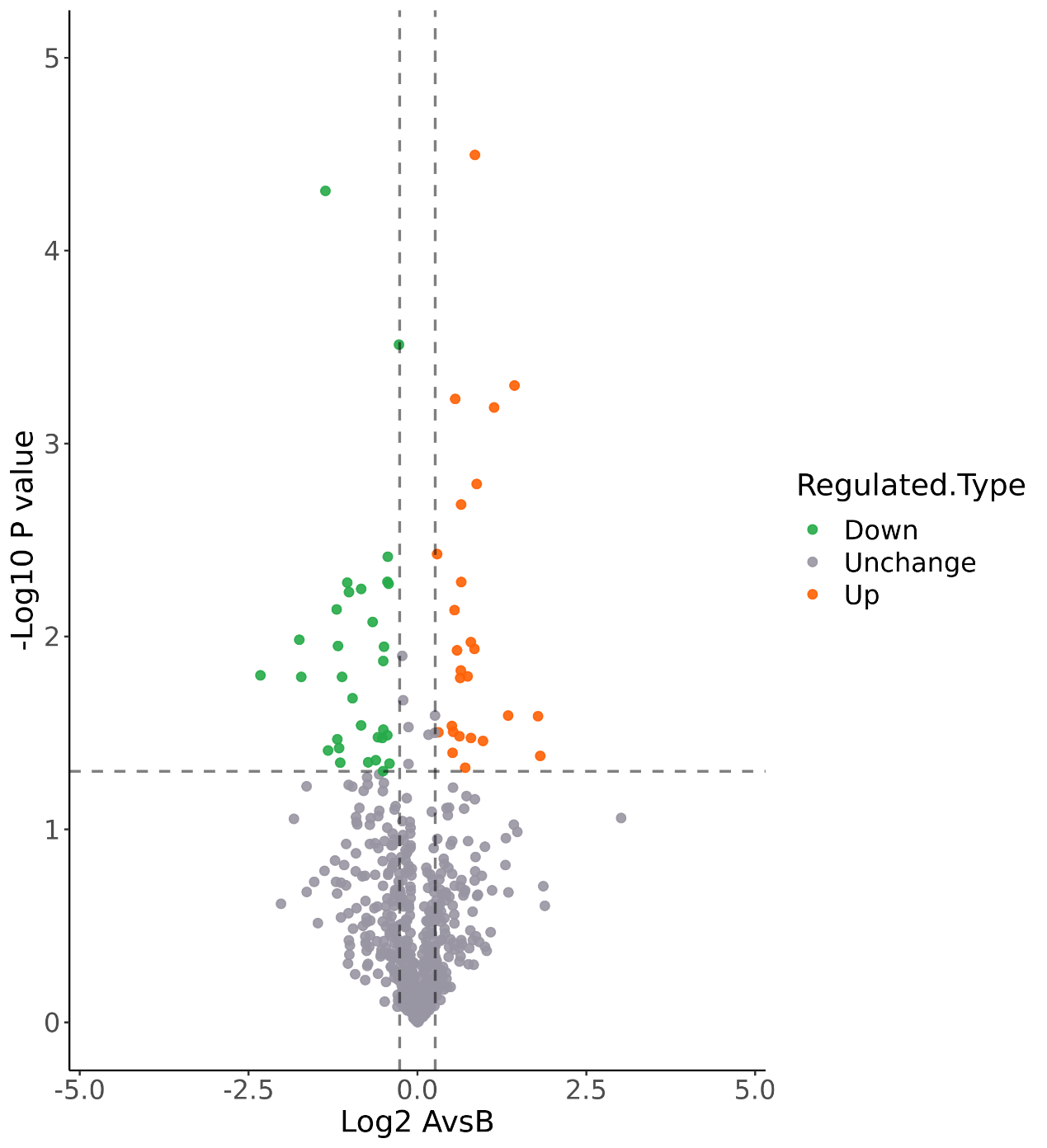


Note: Plasma proteins that underwent significant fold changes (FCs) between Group A and Group B (FC >1.2 or FC <0.8; unpaired two-sided Welch’s t test; p <0.05).

Supplementary figure S4. Feature analysis


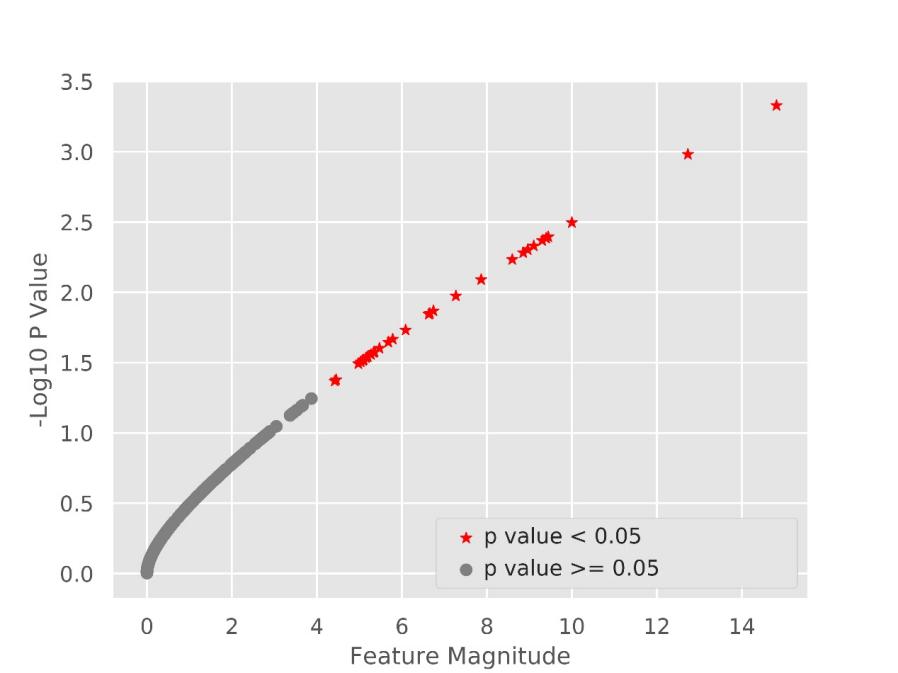


Note: In this figure, X-axis was the score of each expression feature, and Y-axis was the p-value of corresponding expression feature converted to -log10. Among them, the expression characteristics with a p value < 0.05 were indicated in red, and the expression characteristics with a p value > 0.05 were indicated in gray.

Supplementary figure S5. Ranking histogram


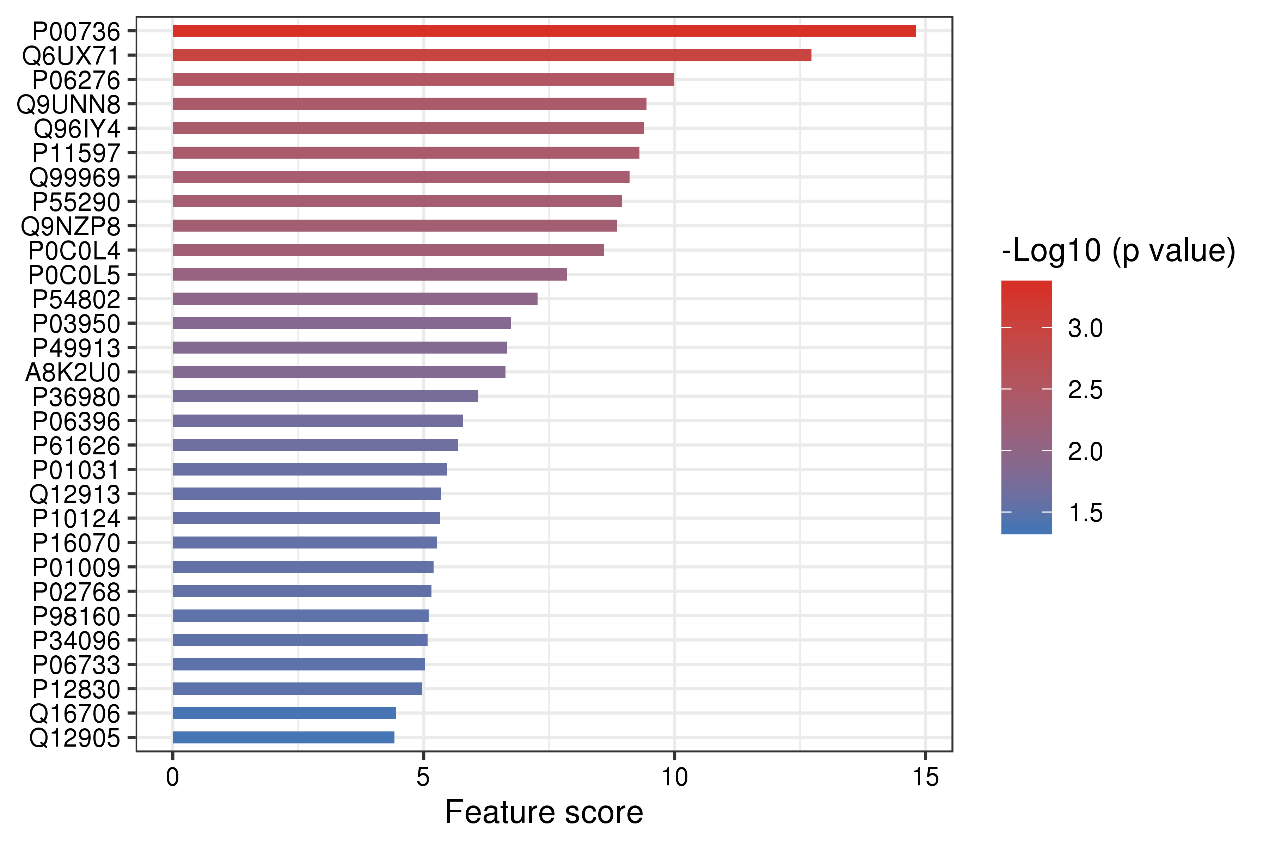


Note: In the figure, the expression feature was sorted according to the score, and the color of the histogram was filled with the feature's -log10 p value.

Supplementary figure S6. Incremental feature selection curve


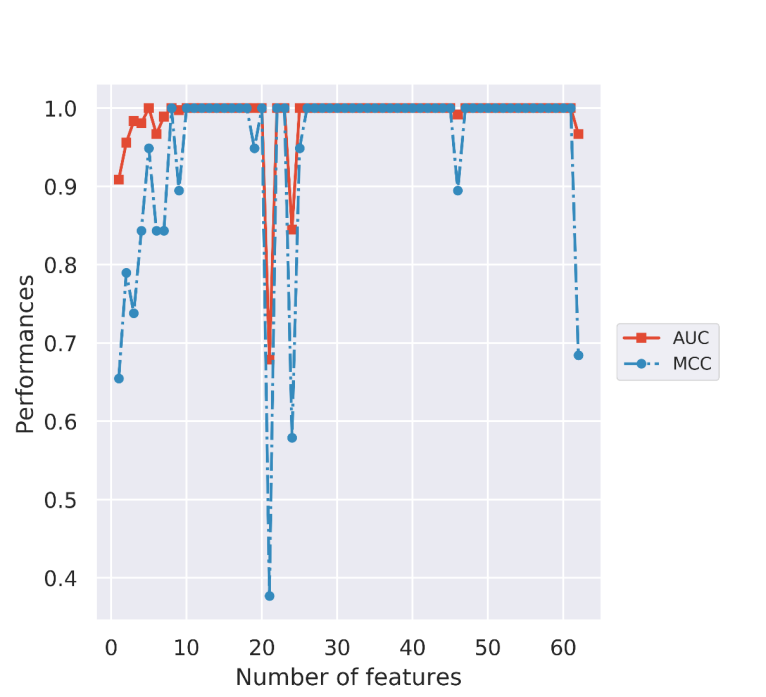


Supplementary table S1**.** Information of candidate proteins selected by machine learning

| **Protein Gene** | **Protein Accession** | **Protein Description** |
| --- | --- | --- |
| C1R | P00736 | Complement C1r subcomponent OS=Homo sapiens OX=9606 GN=C1R PE=1 SV=2 |
| BCHE | P06276 | Cholinesterase OS=Homo sapiens OX=9606 GN=BCHE PE=1 SV=1 |
| CETP | P11597 | Cholesteryl ester transfer protein OS=Homo sapiens OX=9606 GN=CETP PE=1 SV=2 |
| CPB2 | Q96IY4 | Carboxypeptidase B2 OS=Homo sapiens OX=9606 GN=CPB2 PE=1 SV=2 |
| EPCR | Q9UNN8 | Endothelial protein C receptor OS=Homo sapiens OX=9606 GN=PROCR PE=1 SV=1 |
| PLXDC2 | Q6UX71 | Plexin domain-containing protein 2 OS=Homo sapiens OX=9606 GN=PLXDC2 PE=1 SV=1 |

Supplementary figure S7. Box plot of the levels of six candidate proteins in two groups


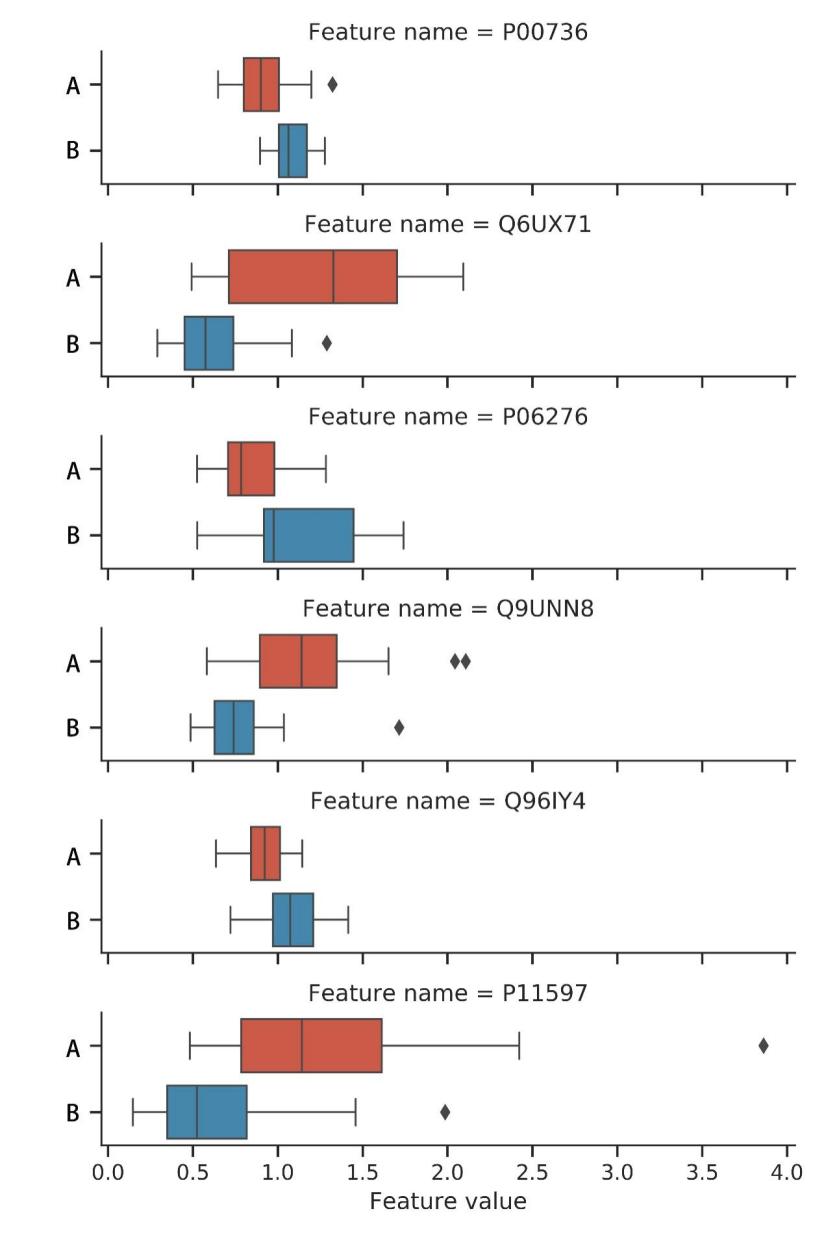


Supplementary figure S8. Expression levels of target proteins (CPB2, EPCR and CETP) in Validation Cohort


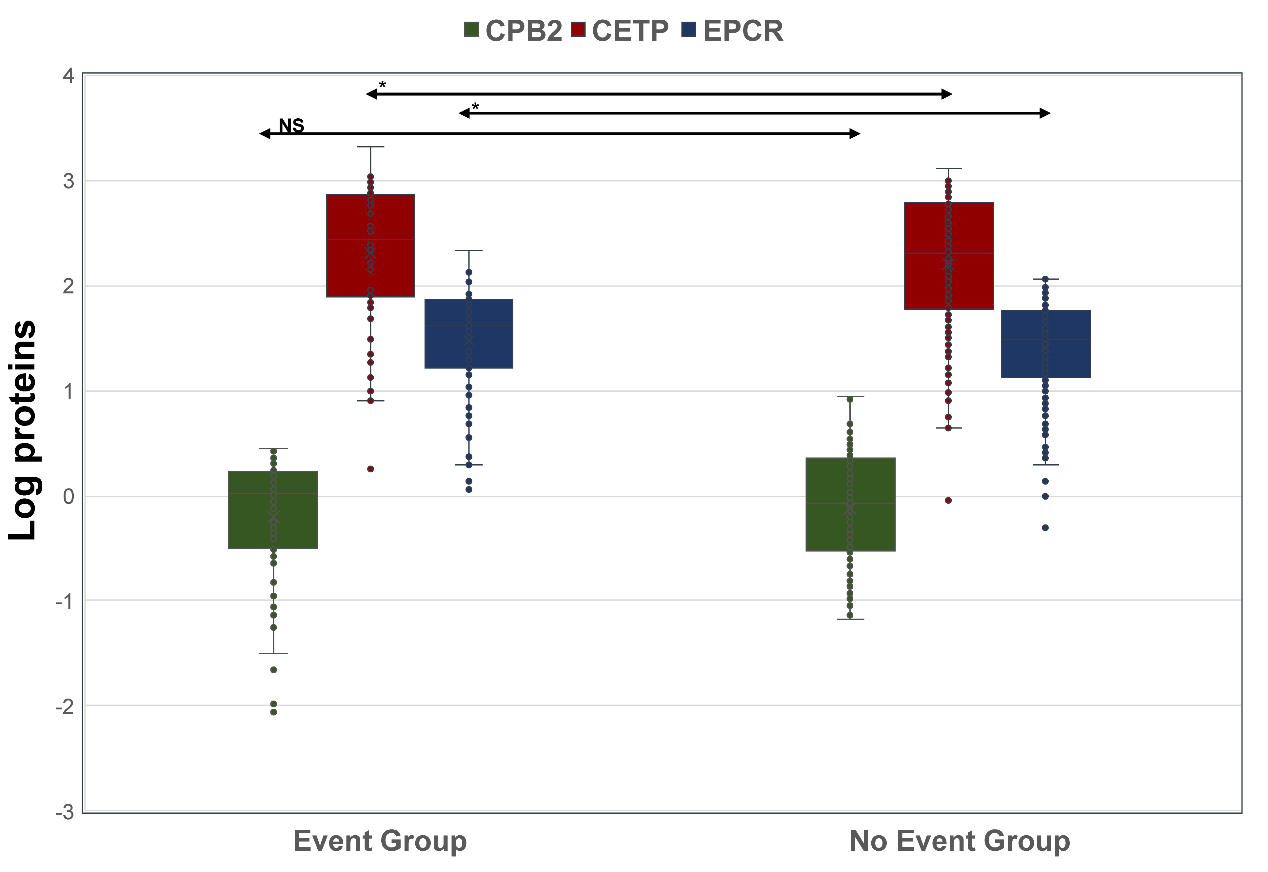


Note: Compared between event group and no event group: *P value < 0.05; NS: No significance.
